# Supplementary material for: Reactivation of previous decisions repulsively biases sensory encoding but attractively biases decision-making
Source: PLoS Biol. 2025 Apr 23;23(4):e3003150. doi: 10.1371/journal.pbio.3003150 (PMC12052181; doi:10.1371/journal.pbio.3003150)
Supplement: S1 Fig — Positive and negative values correspond to aligned and flipped direction, respectively. Horizontal lines denote significant temporal clusters (cluster-based permutation test, p < 0.05, two-sided, corrected). Data supporting this figure can be found at: https://osf.io/c7dwp/. (DOCX) [file pbio.3003150.s002.docx]

**
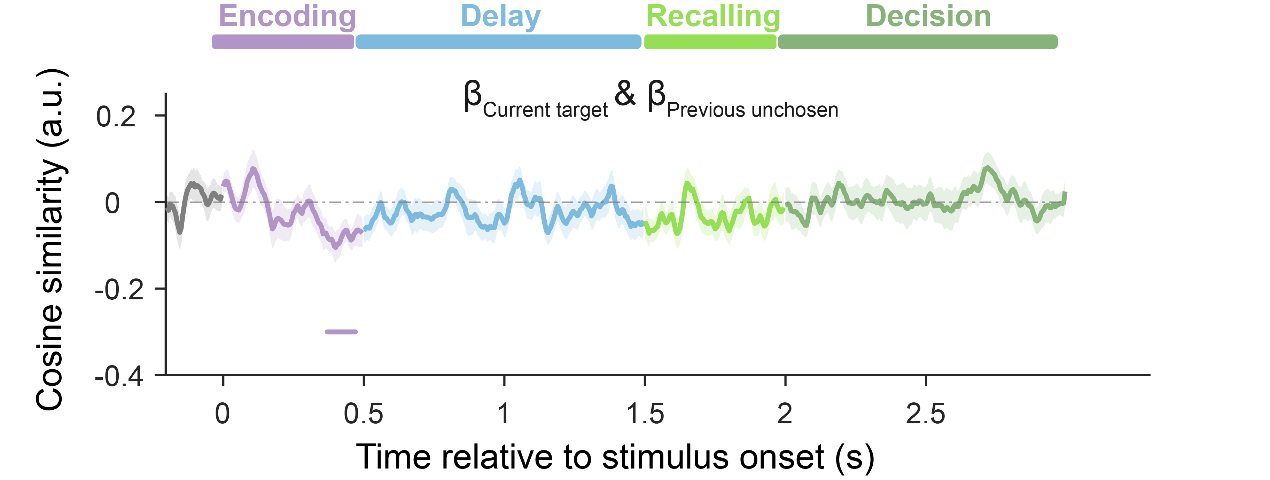
**

**S1 Fig.** **Time-resolved grand average of past-present interactions between current target and previous unchosen location in Experiment 2 (MEG), related to Fig 4**. The shaded areas correspond to ±1 SEM. Positive and negative values correspond to aligned and flipped direction, respectively. Horizontal lines denote significant temporal clusters (cluster-based permutation test, p < 0.05, two-sided, corrected). Data supporting this figure can be found at: https://osf.io/c7dwp/.
