# Supplementary material for: Reactivation of previous decisions repulsively biases sensory encoding but attractively biases decision-making
Source: PLoS Biol. 2025 Apr 23;23(4):e3003150. doi: 10.1371/journal.pbio.3003150 (PMC12052181; doi:10.1371/journal.pbio.3003150)
Supplement: S2 Fig — (A). Cross-subject correlation between serial dependence behavior (x-axis; regression coefficient) and past–present neural interaction (y-axis; averaged within significant clusters) induced by previous chosen location during Encoding (purple) and Decision-making (blue) stages. (B). The same as A but for serial dependence behavior and past–present neural interaction induced by previous unchosen location during Encoding stage. Each dot represents individual participant. The solid lines correspond to the best linear fitting, and the shaded areas correspond to 95% confidence interval. Data supporting this figure can be found at: https://osf.io/c7dwp/. (DOCX) [file pbio.3003150.s003.docx]

**
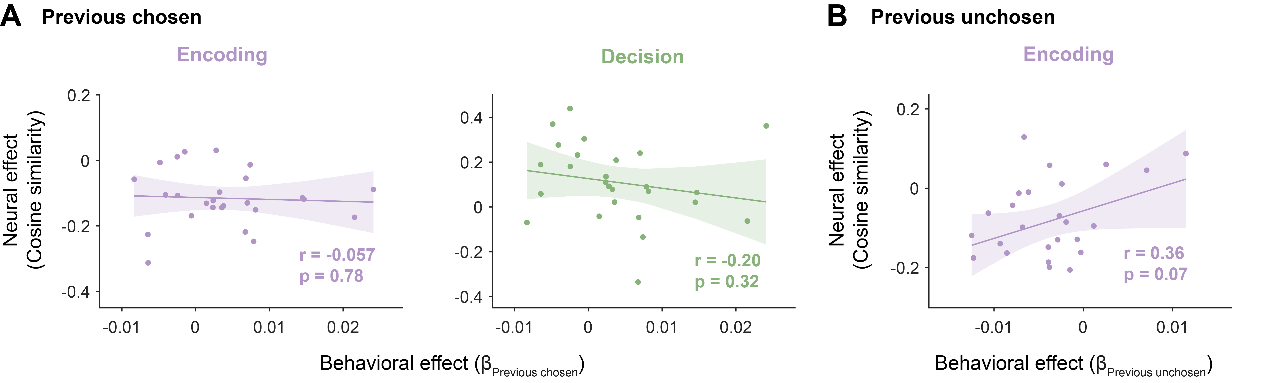
**

**S2 Fig**. **Across-participant neuro-behavioral** **correlation in Experiment 2 (MEG), related to Fig 4. A**. Cross-subject correlation between serial dependence behavior (x-axis; regression coefficient) and past-present neural interaction (y-axis; averaged within significant clusters) induced by previous chosen location during Encoding (purple) and Decision-making (blue) stages. **B**. The same as A but for serial dependence behavior and past-present neural interaction induced by previous unchosen location during Encoding stage. Each dot represents individual participant. The solid lines correspond to the best linear fitting, and the shaded areas correspond to 95% confidence interval. Data supporting this figure can be found at: https://osf.io/c7dwp/.
