# Supplementary material for: Reactivation of previous decisions repulsively biases sensory encoding but attractively biases decision-making
Source: PLoS Biol. 2025 Apr 23;23(4):e3003150. doi: 10.1371/journal.pbio.3003150 (PMC12052181; doi:10.1371/journal.pbio.3003150)
Supplement: S3 Fig — (A). Time-resolved grand averaged decoding results of previous chosen location during encoding stage. The regions of significant temporal clusters displayed on the inflated cortical surface and their names are shown at the leftmost. Dark and light colors denote temporal clusters with or without spatial correction. Except Cuneus (shown in Fig 5), time courses of all regions with significant temporal clusters are plotted. (B). Same as A but for the past–present interactions. (C,D). Same as AB but during decision-making stages. Panel C does not include the result of medial OFC and panel D does not include pars orbitalis. The shaded areas correspond to ±1 SEM. Color-coded horizontal lines denote significant temporal clusters (cluster-based permutation test, p < 0.05, two-sided, corrected). Data supporting this figure can be found at: https://osf.io/c7dwp/. (DOCX) [file pbio.3003150.s004.docx]

**
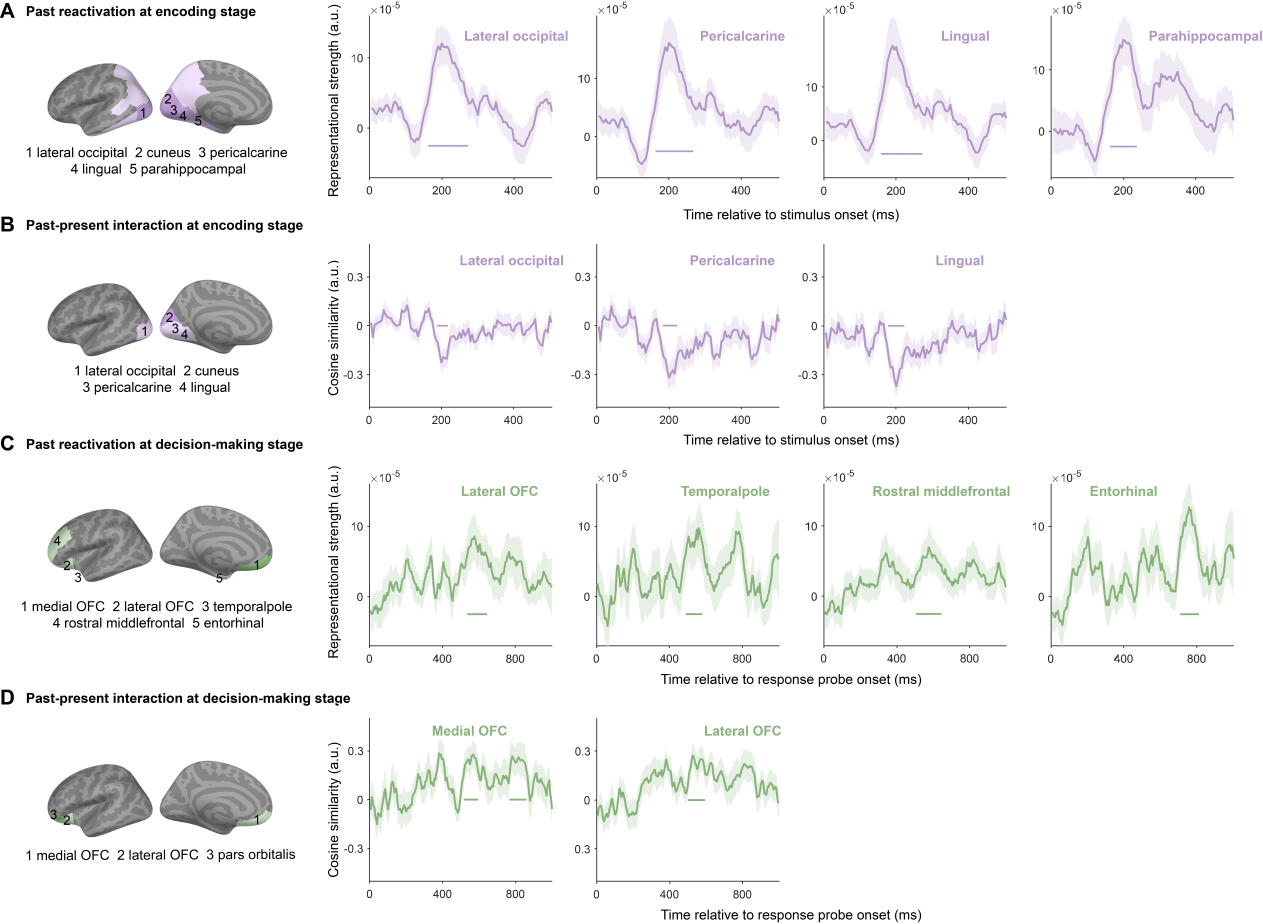
**

**S3 Fig**. **Time courses of two-stage cortical origins of serial biases in all significant regions in Experiment 2 (MEG), related to Fig 5.** **A.** Time-resolved grand averaged decoding results of previous chosen location during encoding stage. The regions of significant temporal clusters displayed on the inflated cortical surface and their names are shown at the leftmost. Dark and light colors denote temporal clusters with or without spatial correction. Except Cuneus (shown in Figure 5), time courses of all regions with significant temporal clusters are plotted. **B.** Same as A but for the past-present interactions. **CD.** Same as AB but during decision-making stages. Panel C does not include the result of medial OFC and panel D does not include pars orbitalis. The shaded areas correspond to ±1 SEM. Color-coded horizontal lines denote significant temporal clusters (cluster-based permutation test, p < 0.05, two-sided, corrected). Data supporting this figure can be found at: https://osf.io/c7dwp/.
