# Supplementary material for: Reactivation of previous decisions repulsively biases sensory encoding but attractively biases decision-making
Source: PLoS Biol. 2025 Apr 23;23(4):e3003150. doi: 10.1371/journal.pbio.3003150 (PMC12052181; doi:10.1371/journal.pbio.3003150)
Supplement: S4 Fig — The grey lines indicate the best linear fitting for each subject and the black line is the best fitting across all subjects. Data supporting this figure can be found at: https://osf.io/c7dwp/. (DOCX) [file pbio.3003150.s005.docx]

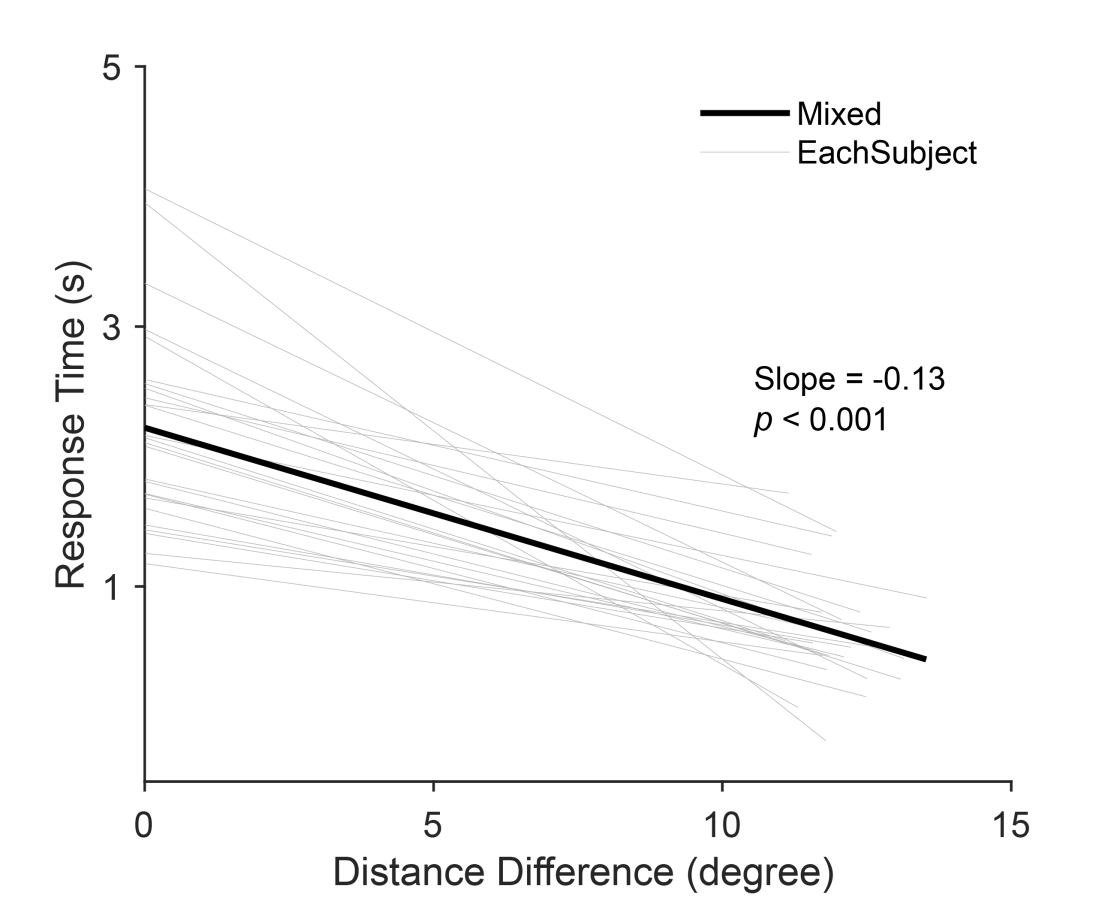


**S4 Fig. Reaction time (Y-axis) as a function of distance difference (X-axis) in Experiment 2, related to Methods**. The grey lines indicate the best linear fitting for each subject and the black line is the best fitting across all subjects. Data supporting this figure can be found at: https://osf.io/c7dwp/.
