# Supplementary material for: Reactivation of previous decisions repulsively biases sensory encoding but attractively biases decision-making
Source: PLoS Biol. 2025 Apr 23;23(4):e3003150. doi: 10.1371/journal.pbio.3003150 (PMC12052181; doi:10.1371/journal.pbio.3003150)
Supplement: S5 Fig — Using leave-one-out method, we trained a linear decoder to reconstruct present locations by employing a multiple linear regression model and tested whether it could be predicted by previous reported location. The neural bias is characterized by the regression coefficient. (A) Time-resolved grand averaged neural bias in Experiment 1. Location reconstruction is based on neural activities on all EEG sensors. (B) Time-resolved grand averaged neural bias in Experiment 2 (sensor level). Location reconstruction is based on neural activities from all MEG sensors. (C,D) Time-resolved grand averaged neural bias during encoding in Cuneus and decision-making in Pars orbitalis. The shaded areas correspond to ±1 SEM. Color-coded horizontal lines denote significant temporal clusters (cluster-based permutation test, p < 0.05, one-sided, corrected). Data supporting this figure can be found at: https://osf.io/c7dwp/. (DOCX) [file pbio.3003150.s006.docx]

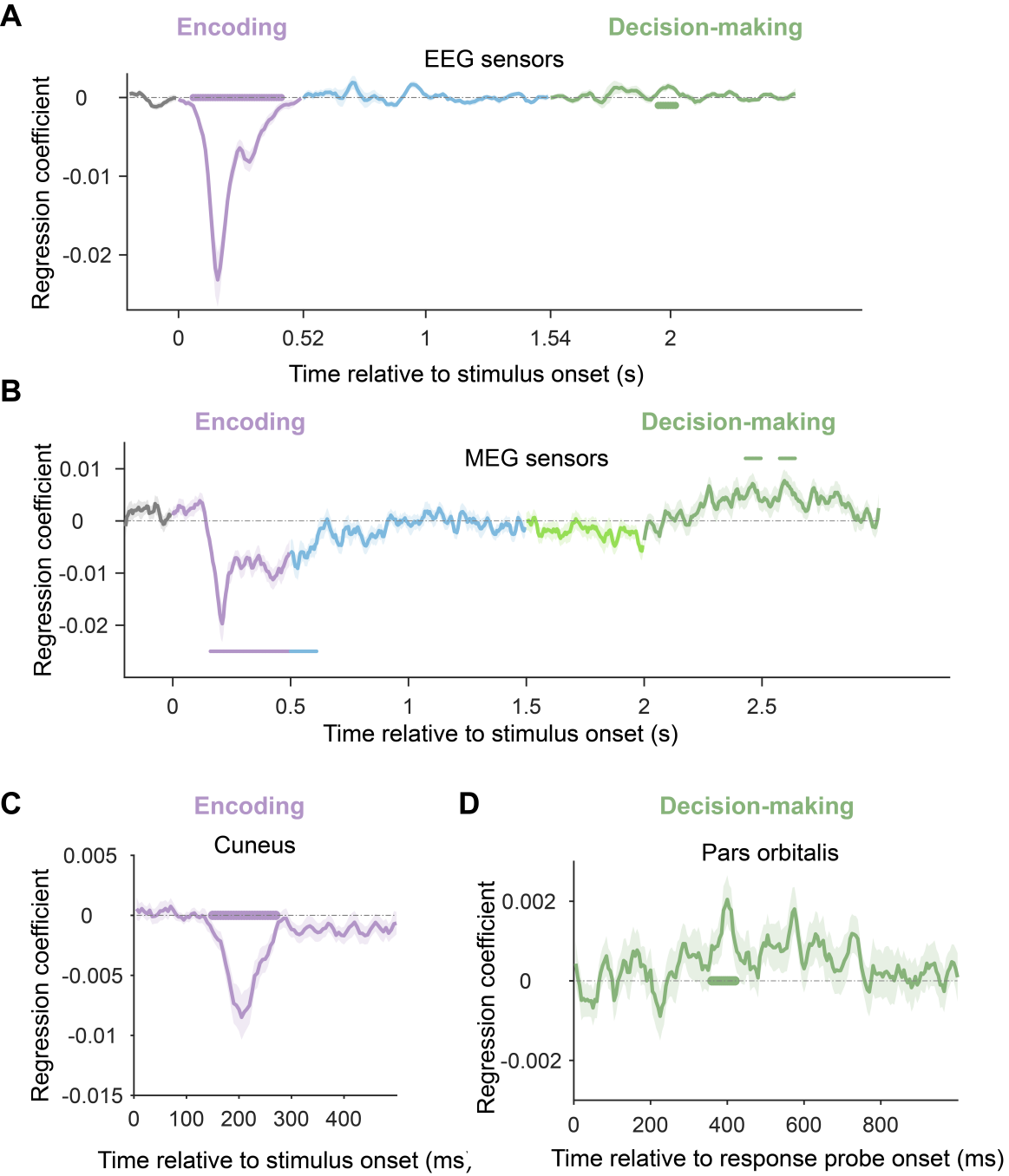


**S5 Fig. Neural bias in present location representations caused by previous reported location**. Using leave-one-out method, we trained a linear decoder to reconstruct present locations by employing a multiple linear regression model and tested whether it could be predicted by previous reported location. The neural bias is characterized by the regression coefficient. **A. Time-resolved grand averaged neural bias in Experiment 1.** Location reconstruction is based on neural activities on all EEG sensors. **B.** **Time-resolved grand averaged neural bias in Experiment 2 (sensor level).** Location reconstruction is based on neural activities from all MEG sensors. **CD. Time-resolved grand averaged neural bias during encoding in Cuneus and decision-making in Pars orbitalis.** The shaded areas correspond to ±1 SEM. Color-coded horizontal lines denote significant temporal clusters (cluster-based permutation test, p < 0.05, one-sided, corrected).Data supporting this figure can be found at: https://osf.io/c7dwp/.
