# Supplementary material for: Reactivation of previous decisions repulsively biases sensory encoding but attractively biases decision-making
Source: PLoS Biol. 2025 Apr 23;23(4):e3003150. doi: 10.1371/journal.pbio.3003150 (PMC12052181; doi:10.1371/journal.pbio.3003150)
Supplement: S6 Fig — When decoding information in the next trial, we regressed out the influence of current locations from neural signals and used the residuals. The shaded areas correspond to ±1 SEM. Color-coded horizontal lines denote significant temporal clusters (cluster-based permutation test, p < 0.05, two-sided, corrected). Data supporting this figure can be found at: https://osf.io/c7dwp/. (DOCX) [file pbio.3003150.s007.docx]

**
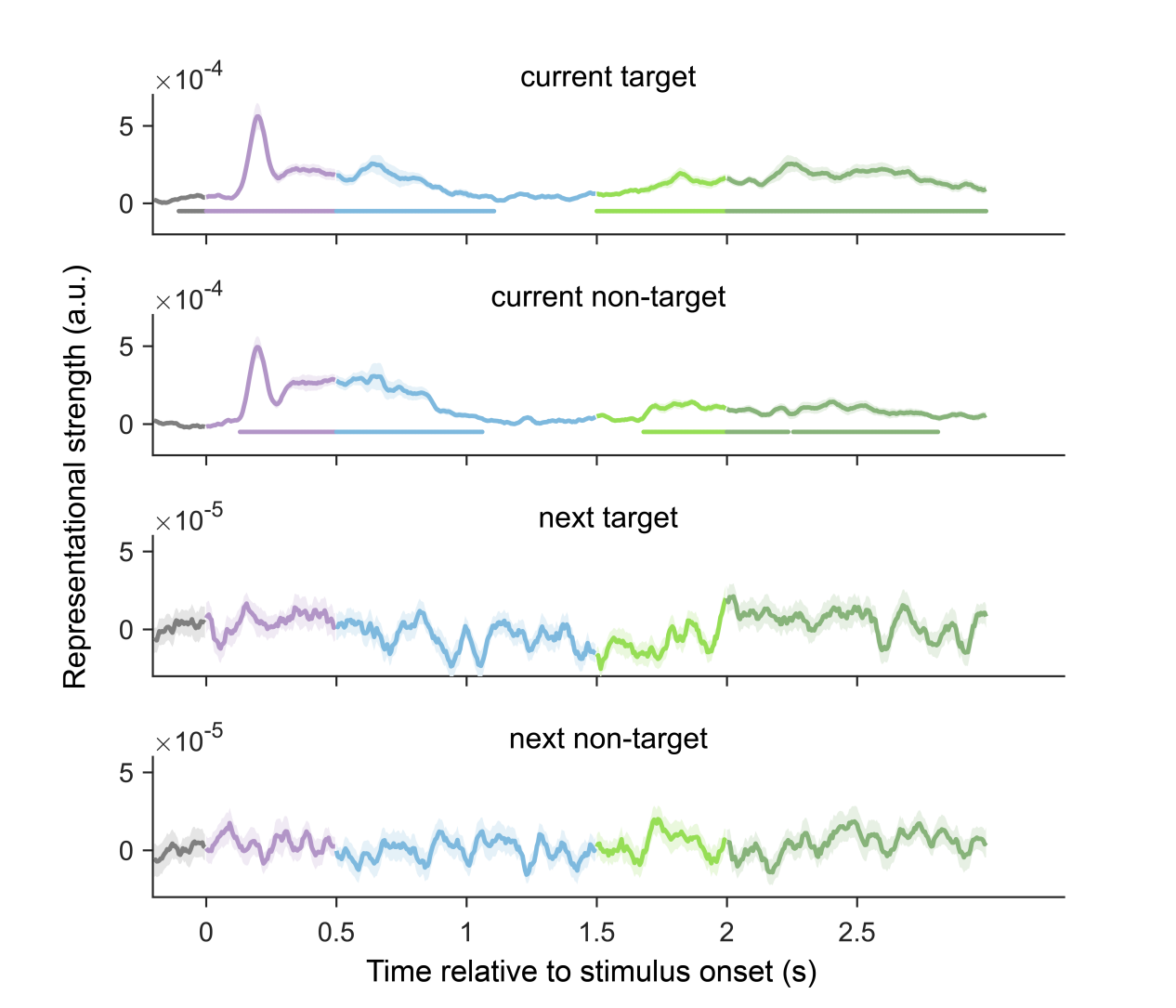
S6 Fig. Neural decoding of information in the current and next trials (Experiment 2).** When decoding information in the next trial, we regressed out the influence of current locations from neural signals and used the residuals. The shaded areas correspond to ±1 SEM. Color-coded horizontal lines denote significant temporal clusters (cluster-based permutation test, p < 0.05, two-sided, corrected). Data supporting this figure can be found at: https://osf.io/c7dwp/.
